# FliH and FliI ensure efficient energy coupling of flagellar type III protein export in *Salmonella*

**DOI:** 10.1002/mbo3.340

**Published:** 2016-02-25

**Authors:** Tohru Minamino, Miki Kinoshita, Yumi Inoue, Yusuke V. Morimoto, Kunio Ihara, Satomi Koya, Noritaka Hara, Noriko Nishioka, Seiji Kojima, Michio Homma, Keiichi Namba

**Affiliations:** ^1^ Graduate School of Frontier BiosciencesOsaka University1‐3 YamadaokaSuitaOsaka565‐0871Japan; ^2^ Quantitative Biology CenterRIKEN6‐2‐3 FuruedaiSuitaOsaka565‐0874Japan; ^3^ Center for Gene ResearchNagoya UniversityChikusa‐kuNagoya464‐8602Japan; ^4^ Departments of Food Science and NutritionFaculty of Human life and ScienceDoshisha Women's College of Liberal ArtsKyoto602‐0893Japan; ^5^ Division of Biological ScienceGraduate School of ScienceNagoya UniversityChikusa‐kuNagoya464‐8602Japan

**Keywords:** Bacterial flagella, cross‐complementation, FlhA, mutational robustness, Type III protein export

## Abstract

For construction of the bacterial flagellum, flagellar proteins are exported via its specific export apparatus from the cytoplasm to the distal end of the growing flagellar structure. The flagellar export apparatus consists of a transmembrane (TM) export gate complex and a cytoplasmic ATPase complex consisting of FliH, FliI, and FliJ. FlhA is a TM export gate protein and plays important roles in energy coupling of protein translocation. However, the energy coupling mechanism remains unknown. Here, we performed a cross‐complementation assay to measure robustness of the energy transduction system of the export apparatus against genetic perturbations. *Vibrio* FlhA restored motility of a *Salmonella* Δ*flhA* mutant but not that of a Δ*fliH‐fliI flhB(P28T)* Δ*flhA* mutant. The *flgM* mutations significantly increased flagellar gene expression levels, allowing *Vibrio* FlhA to exert its export activity in the Δ*fliH‐fliI flhB(P28T)* Δ*flhA* mutant. Pull‐down assays revealed that the binding affinities of *Vibrio* FlhA for FliJ and the FlgN–FlgK chaperone–substrate complex were much lower than those of *Salmonella* FlhA. These suggest that *Vibrio* FlhA requires the support of FliH and FliI to efficiently and properly interact with FliJ and the FlgN–FlgK complex. We propose that FliH and FliI ensure robust and efficient energy coupling of protein export during flagellar assembly.

## Introduction

The bacterial flagellum is a macromolecular assembly made of about 30 different proteins with their copy numbers ranging from a few to tens of thousands and consists of the basal body rings and a tubular axial structure. For assembly of the flagellar axial structure beyond the cell membranes, flagellar axial proteins are exported by a type III export apparatus from the cytoplasm to the distal end of the growing structure. The export apparatus consists of a transmembrane (TM) export gate complex made of FlhA, FlhB, FliO, FliP, FliQ, and FliR, and a cytoplasmic ATPase complex consisting of FliH, FliI, and FliJ (Macnab 2003; Chevance and Hughes 2008; Minamino et al. 2008; Minamino 2014). The export apparatus requires both ATP and proton motive force (PMF) across the cytoplasmic membrane as the fuels for rapid and efficient protein export (Minamino and Namba 2008; Paul et al. 2008). These component proteins are highly homologous to those of the type III secretion systems of pathogenic bacteria, which inject virulence effector proteins into their eukaryotic host cells for invasion (Cornelis 2006).

FlhA consists of an N‐terminal integral membrane domain with eight predicted TM helices (FlhA_TM_), a flexible linker (FlhA_L_), and a C‐terminal cytoplasmic domain (FlhA_C_) (Fig. S1) (Minamino et al. 1994). FlhA_TM_ is responsible for the interaction with the basal body MS ring protein FliF (Kihara et al. 2001). A highly conserved hydrophilic cytoplasmic loop between TM‐4 and TM‐5 is responsible for the interaction of FlhA with FliR (Hara et al. 2011). FlhA_C_ provides binding sites for FliH, FliI, FliJ, flagellar type III secretion chaperones, and export substrates (Minamino and Macnab 2000a; Minamino et al. 2003, 2009, 2010, 2011, 2012a; Bange et al. 2010; Kinoshita et al. 2013). FlhA_C_ consists of four subdomains, D1, D2, D3, and D4 (Saijo‐Hamano et al. 2010). A well‐conserved hydrophobic dimple located at the interface of domains D1 and D2 of FlhA_C_ is responsible for interactions of FlhA with the FlgN/FlgK, FlgN/FlgL, FliT/FliD, and FliS/FliC chaperone–substrate complexes (Bange et al. 2010; Minamino et al. 2012a; Kinoshita et al. 2013). A highly conserved Phe‐459 residue in this hydrophobic dimple of FlhA_C_ is critical for the interaction with a well‐conserved Tyr residue of FlgN, FliT, and FliS chaperones (Minamino et al. 2012a; Kinoshita et al. 2013). The G368C mutation in domain D1 of FlhA_C_ induces a large conformational change in domain D2 at the restrictive temperature of 42°C, thereby blocking the export process after the FliH–FliI–FliJ–export substrate complex binds to the FlhA–FlhB docking platform (Minamino et al. 2010; Shimada et al. 2012). This suggests that the D2 domain is directly involved in the translocation of the export substrate into the central channel of the growing flagellar structure (Shimada et al. 2012). FlhA_L_ is involved in an interaction between FlhA and FliJ (Bange et al. 2010; Minamino et al. 2011). FliH and FliI help FliJ to efficiently and properly interact with FlhA_L_, thereby fully activating the PMF‐driven export gate (Minamino et al. 2011; Ibuki et al. 2013). ATP hydrolysis by FliI is linked to the gate‐activation process (Minamino et al. 2014). These observations suggest that FlhA plays important roles in energy coupling of flagellar type III protein export. However, the energy coupling mechanism remains unclear.

To examine robustness of the energy coupling mechanism of flagellar type III protein export against genetic perturbations, we replaced the *Salmonella enterica flhA* gene (*StflhA*) by the *flhA* gene of *Vibrio alginolyticus* (*VaflhA*). We show that VaFlhA exerts its export activity in *S. enterica* in the presence of FliH and FliI, but not in their absence. We also show that VaFlhA requires FliH and FliI for efficient interactions with FliJ and the FlgN–FlgK chaperone–substrate complex.

## Experimental Procedures

### **Bacteria, plasmids, P22‐mediated transduction, DNA manipulations, and media**

*Salmonella* strains and plasmids used in this study are listed in Table 1. P22‐mediated transduction was carried out as described (Yamaguchi et al. ). L‐broth (LB) and soft tryptone agar plates were prepared as described previously (Minamino and Macnab 1999, 2000a). To construct a *Salmonella Vibrio flhA* strain*, the flhA* gene on the chromosome was replaced by the *Vibrio flhA* allele using the *λ* Red homologous recombination system (Datsenko and Wanner 2000). Ampicillin and kanamycin were added at a final concentration of 100 and 15 *μ*g/mL, respectively, if needed.

**Table 1:** Strains and Plasmids used in this study

Strains and plasmids	Relevant characteristics	Source or reference
*E. coli*		
BL21(DE3)	Overexpression of proteins	Novagen
*Salmonella*		
SJW1103	Wild‐type for motility and chemotaxis	Yamaguchi et al. (1984)
SJW1368	∆*cheW‐flhD*	Ohnishi et al. (1992, 1994)
NH001	∆*flhA*	Hara et al. (2011)
NH002	∆*flhA flhB(P28T)*	Hara et al. (2011)
NH004	∆*fliH‐fliI* ∆*flhA flhB(P28T)*	Hara et al. (2011)
MMHI0117	∆*fliH‐fliI flhB(P28T)*	Minamino and Namba (2008)
MMB017	*flhB(P28T)*	Minamino and Namba (2008)
MMA2001	*VaflhA*	This study
MMA2002	∆*fliH‐fliI* ∆*flhA flhB(P28T) flgM/*pNY101	This study
MMA2003	∆*fliH‐fliI* ∆*flhA flhB(P28T) flgM/*pNY101	This study
MMA2004	∆*fliH‐fliI* ∆*flhA flhB(P28T) flgM/*pNY101	This study
Plasmids		
pGEX‐6p‐1	Expression vector	GE Healthcare
pSU41	Expression vector	Bartolomé et al. (1991)
pTrc99AFF4	Expression vector	Ohnishi et al. (1997)
pHMK215	pET3c/FlgN	Minamino et al. (2012b)
pMKGK2	pTrc99A/FlgK	Furukawa et al. (2002)
pMM130	pTrc99AFF4/FlhA	Kihara et al. (2001)
pMMHA1001	pGEX6p‐1/GST‐FlhA_C_	Minamino et al. (2009)
pMM306	pTrc99A/His‐FliH	Minamino and Macnab (2000a, 2000b)
pMM406	pTrc99A/His‐FliJ	Minamino and Macnab (2000a, 2000b)
pMM1702	pTrc99A/His‐FliI	Minamino and Macnab (2000a, 2000b)
pMKMHA003	pTrc99AFF4/VaFlhA_TM‐L_‐StFlh_C_	This study
pMKMHA004	pTrc99AFF4/VaFlhA_TM_‐StFlhA_L‐C_	This study
pMKM005	pGEX6p‐1/GST‐VaFlhA_C_	This study
pNY101	pSU41/VaFlhA	This study

### DNA manipulations

DNA manipulations were carried out as described (Saijo‐Hamano et al. 2004). DNA sequencing reactions were carried out using BigDye v3.1 as described in the manufacturer's instructions (Applied Biosystems, Tokyo, Japan), and then the reaction mixtures were analyzed by a 3130 Genetic Analyzer (Applied Biosystems).

### Whole‐genome sequencing and data analysis

Genomic DNAs were isolated from the *fliH‐fliI flhB*(*P28T) VaflhA* strain and its pseudorevertants. Nextera XT kits (Illumina, Tokyo, Japan) were used to generate a MiSeq compatible library from the *Salmonella* genomic DNA. The constructed libraries were then loaded into a MiSeq 600‐Cycle v3 Reagent Kit (Illumina). The fastq files produced by the MiSeq were imported to a CLC Genomic work bench (Qiagen, Boston, MA, USA) and SNPs were detected using traditional variants detection tool by comparing the parent strain and their pseudorevertants.

### Motility assay

Fresh transformants were inoculated onto soft tryptone agar plates and incubated at 30°C. At least seven independent assays were performed.

### Secretion assay

Cells were grown at 30°C with shaking until the cell density had reached an OD_600_ of ca. 1.2–1.4. To test the effect of carbonyl cyanide *m*‐chlorophenylhydrazone (CCCP) on flagellar protein export, the cells were grown with shaking in 5 mL of LB containing ampicillin at 30°C until the cell density had reached an OD_600_ of ca. 0.6–0.7. After washing twice with LB, the cells were resuspended in 5 mL LB with or without CCCP and incubated at 30°C for 1 h. Cultures were centrifuged to obtain the cell pellets and culture supernatants. To test the effect of 100 mmol/L NaCl on flagellar protein export, the cells were grown with shaking in 5 mL of LB with or without 100 mmol/L NaCl at 30°C until the cell density had reached an OD_600_ of ca. 1.2–1.4. After centrifugation, the whole cellular and culture supernatant fractions were collected separately. Cell pellets were resuspended in sodium dodecyl sulfate (SDS)‐loading buffer normalized by the cell density to give a constant amount of cells. The proteins in the culture supernatants were precipitated by 10% trichloroacetic acid, suspended in a Tris‐SDS loading buffer, and heated at 95°C for 3 min. After SDS‐polyacrylamide gel electrophoresis (PAGE), immunoblotting with polyclonal anti‐FlgD, anti‐FlgE, anti‐FlgK, anti‐FlgL, anti‐FliD, and anti‐FliK antibodies was carried out as described previously (Minamino and Macnab 1999). Detection was done with an ECL plus immunoblotting detection kit (GE Healthcare, Tokyo, Japan). At least three independent experiments were carried out.

### Observation of flagellar filaments with a fluorescent dye

The flagellar filaments produced by *Salmonella* cells were labeled using anti‐FliC antiserum and anti‐rabbit IgG conjugated with Alexa Fluor^®^ 594 (Invitrogen) as described (Minamino et al. 2014). The cells were observed by fluorescence microscopy as described previously (Morimoto et al. 2010). Fluorescence images were analyzed using ImageJ software version 1.48 (National Institutes of Health, USA).

### Protein expression and purification

The soluble fractions prepared from SJW1368 carrying pMMHA1001 or pMKMHA005 were loaded onto a glutathione Sepharose 4B column (GE Healthcare). After washing with PBS (8 g of NaCl, 0.2 g of KCl, 3.63 g of Na_2_HPO_4_·12H_2_O, 0.24 g of KH_2_PO_4_, pH 7.4 per liter), proteins were eluted with 50 mmol/L Tris‐HCl, pH 8.0, 10 mmol/L reduced glutathione. Fractions containing glutathione‐S‐transferase (GST)‐tagged proteins were pooled and dialyzed overnight against phosphate buffered saline (PBS) at 4°C with three changes of PBS.

His‐FliH, His‐FliI, and His‐FliJ were overproduced in SJW1368 transformed with pMM306, pMM1702, and pMM406, respectively, and purified by Ni‐NTA affinity chromatography as described previously (Minamino and Macnab 2000a).

FlgK and FlgN were overproduced in BL21 (DE3) transformed with pMKGK2, and pHMK215, respectively, and purified as described previously (Furukawa et al. 2002; Minamino et al. 2012a).

### Pull‐down assay using GST affinity chromatography

Purified His‐FliI, His‐FliH, His‐FliJ, and FlgN/FlgK complex was mixed GST‐*Salmonella* FlhA_C_ or GST‐*Vibrio* FlhA_C_, and then the mixtures were dialyzed overnight against PBS at 4°C with three changes of PBS. These mixtures were loaded onto a glutathione Sepharose 4B column (bed volume, 1 mL) pre‐equilibrated with 20 mL of PBS and washed with 10 mL of PBS at a flow rate of ca. 0.5 mL/min. Bound proteins were eluted with 5 mL of 50 mmol/L Tris‐HCl, pH 8.0, 10 mmol/L reduced glutathione. Eluted fractions were analyzed by SDS‐PAGE with Coomassie Brilliant blue (CBB) staining and immunoblotting. At least three independent experiments were carried out.

### Sequence alignment

Sequence alignment was carried out by Clustal Omega (http://www.ebi.ac.uk/Tools/msa/clustalo/).

## Results

### *Vibrio* FlhA restores motility of a *Salmonella flhA* null mutant

**Figure 1:**
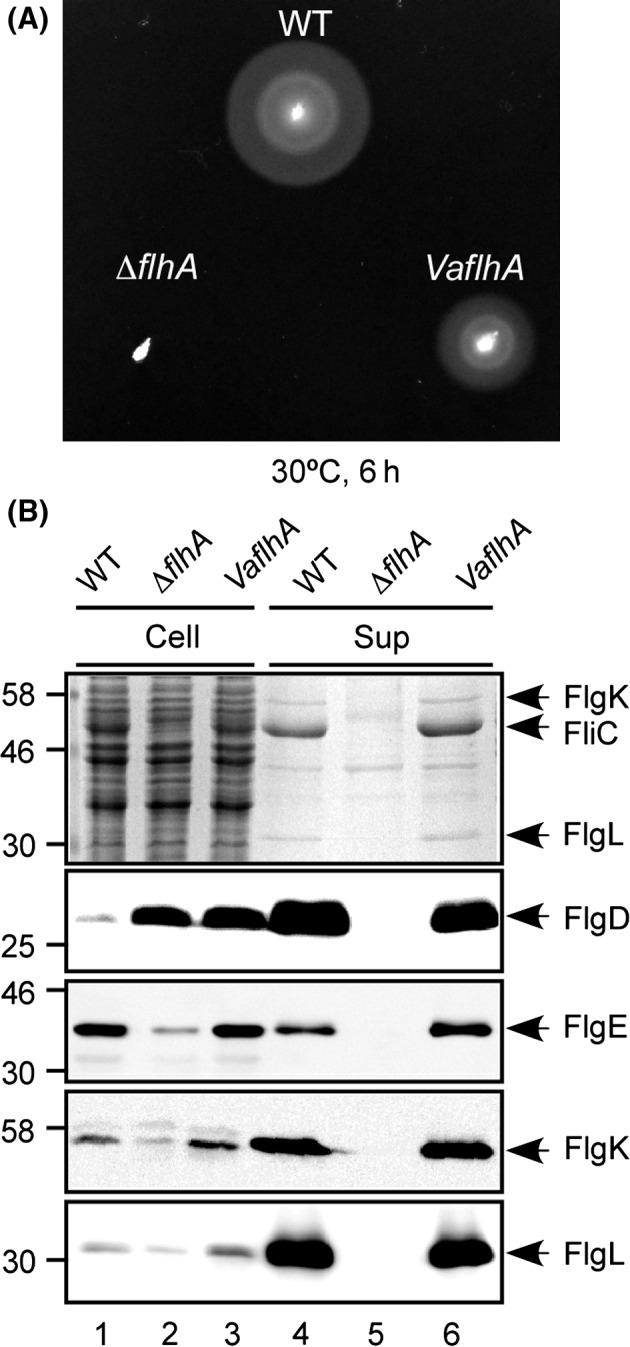
Cross‐complementation assay of *Vibrio* FlhA. (A) Motility of NH001 transformed with pNY101 (*VaflhA*) in soft agar. SJW1103 carrying pSU41 (WT) and NH001 harboring pSU41 (∆*flhA*) are used as the positive and negative controls. Plates were incubated at 30°C for 6 h. (B) Secretion assays. Whole‐cell proteins (Cell) and culture supernatant fractions (Sup) were prepared from the above transformants, and then analyzed by Coomassie Brilliant blue (CBB) staining (first row) and immunoblotting, using polyclonal anti‐FlgD (second row), anti‐FlgE (third row), anti‐FlgK (fourth row), or anti‐FlgL (fifth row) antibody. The positions of molecular mass markers are indicated on the left.

It has been shown that FlhA plays important roles in an energy coupling mechanism of flagellar type III protein export (Minamino et al. 2011). To measure robustness of the energy transduction system of the flagellar type III export apparatus, we performed a cross‐complementation assay of FlhA. *V. alginolyticus* is a *γ*‐proteobacterium that has two circular chromosomal DNAs. Interestingly, *V. alginolyticus* has two distinct sets of the flagellar systems, a polar flagellar system, and a lateral flagellar system, of which genes are encoded on chromosomal DNA I and II, respectively. The polar flagellum is a Na^+^‐driven rotary motor, whereas the lateral flagellum is a H^+^‐driven rotary motor (Zhu et al. 2013). It has been reported that *V. alginolyticus* also has two distinct SecDF complexes, SecDF1 and SecDF2, of which genes are encoded on chromosomal DNA I and II, respectively and that SecDF1 utilizes Na^+^ as the coupling ion to facilitate protein translocation, whereas SecDF2 requires H^+^ instead of Na^+^ (Ishii et al. 2015). *Salmonella* utilizes PMF across the cytoplasmic membrane as the energy source for flagellar motility as well as flagellar type III protein export. Because structural and functional diversities of the bacterial flagellum have been observed among bacterial species although its core structure is highly conserved (Minamino and Imada 2015), we investigated whether the FlhA protein of the Na^+^‐driven polar flagellar system of *V. alginolyticus* (VaFlhA) would be functional in the proton‐driven *Salmonella* flagellar system. The amino acid sequence of VaFlhA has 73.2% similarity and 52.9% identity with the FlhA protein of *S. enterica* serovar Typhimurium (StFlhA) (Fig. S1). We first transformed a *Salmonella flhA* null mutant (Δ*flhA*) with a pSU41‐based plasmid encoding VaFlhA and analyzed the motility of the resulting transformants in soft agar (Fig. 1A). VaFlhA restored motility of the Δ*flhA* mutant in soft agar although not to the *Salmonella* wild‐type level. In agreement with this, immunoblotting analyses revealed that FlgD, FlgE, FlgK, FlgL, and FliC were detected in the culture supernatants (Fig. 1B).

**Figure 2:**
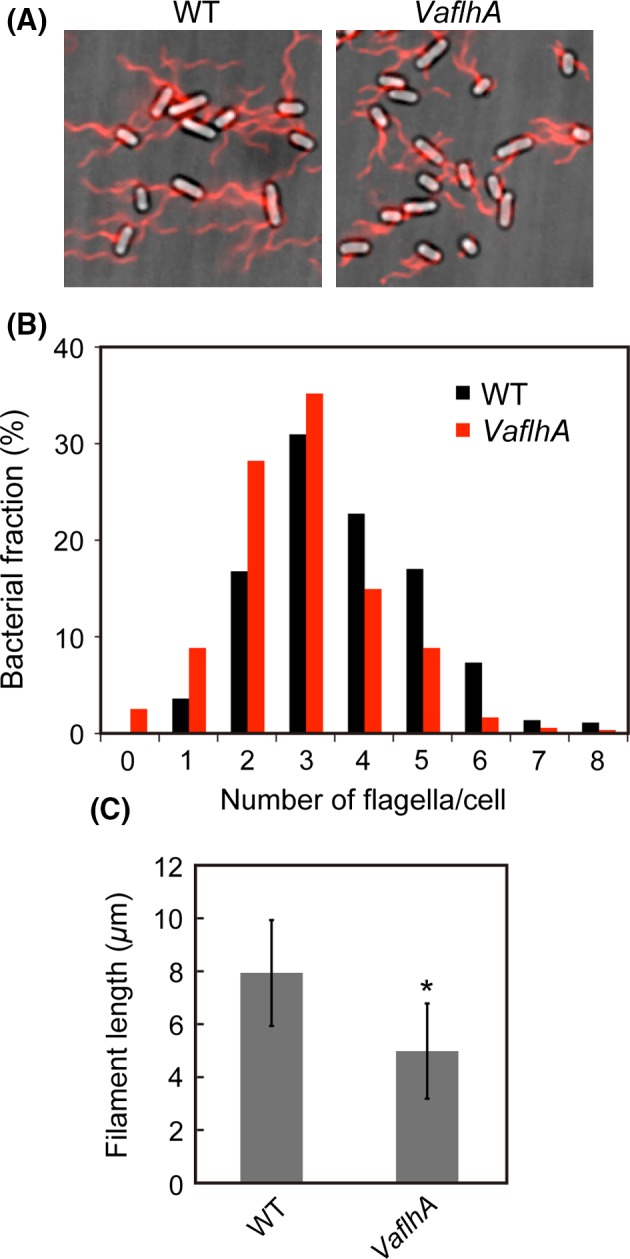
Measurements of the number and length of the flagellar filaments produced by the *VaflhA* cells. (A) Fluorescent images of SJW1103 (WT) and MMA2001 (*VaflhA*). Flagellar filaments were labeled with Alexa Fluor 594. The fluorescence images of the filaments labeled with Alexa Fluor 594 (red) were merged with the bright‐field images of the cell bodies. (B) Distribution of the number of the flagellar filaments in the wild‐type (black) and *VaflhA* cells (red). More than 400 cells for each transformants were counted. (C) Measurements of the length of the flagellar filaments. Filament length is the average of more than 150 cells, and vertical lines are standard deviations. Statistical analysis using Student's *t*‐test shows a significant difference with an asterisk (*P *<* *0.001).

To test if the complementation ability of VaFlhA could be a consequence of its multicopy effect on the motility, the *StflhA* gene on the chromosomal DNA was replaced with the *VaflhA* gene using the *λ* Red homologous recombination system (Datsenko and Wanner 2000). The motility of the *Salmonella VaflhA* strain was lower than that of the *Salmonella* wild‐type strain and essentially the same as that of the *Salmonella* Δ*flhA* mutant transformed with pSU41‐VaFlhA (Fig. S2A). FlgD and FlgE were secreted to the culture supernatant at the wild‐type levels, and FliC, FlgK, and FlgL were secreted less than the wild‐type levels (Fig. S2B, lanes 4 and 6). Consistently, most of the *VaflhA* cells produced flagella at the wild‐type level although the length of flagellar filaments produced by the *VaflhA* cells was shorter than the wild‐type level (Fig. 2). Because the localization of FlhA to the basal body depend on the MS ring protein FliF, a basal body C ring protein FliG, and export gate proteins FliO, FliP, FliQ, and FliR, but not on an export gate protein FlhB, cytoplasmic proteins FliH, FliI, FliJ, and the remaining C ring proteins FliM and FliN (Morimoto et al. 2014), these results indicate that VaFlhA can assemble into the export gate complex within the MS ring with other *Salmonella* gate component proteins to exert its export activity in *Salmonella*.

### *Vibrio* FlhA forms a PMF‐driven export engine

The TM export gate complex made of FlhA, FlhB, FliO, FliP, FliQ, and FliR is powered by PMF across the cytoplasmic membrane (Minamino and Namba 2008; Paul et al. 2008). Therefore, we next tested whether treatment with a protonophore, CCCP, affects flagellar protein export by the *VaflhA* cells (Fig. S3A). The cellular levels of FlgD were maintained even in the presence of 50 *μ*mol/L CCCP (lanes 1–6). However, the levels of FlgD secretion by the wild‐type and *VaflhA* cells both diminished (lanes 8 and 12), indicating that PMF is absolutely essential for FlgD export in the *VaflhA* cells.

The SecDF complex of *Escherichia. coli* utilizes H^+^ as the coupling ion to function in an ATP‐independent stage of protein translocation, whereas the SecDF1 complex of *V. alginolyticus* utilizes Na^+^ as the coupling ion instead of H^+^ even in *E. coli* cells (Tsukazaki et al. 2011; Ishii et al. 2015). Therefore, we tested whether Na^+^ ion affects the secretion rate of FlgD by the *VaflhA* cells (Fig. S3B). The FlgD secretion levels by the wild‐type and *VaflhA* cells both showed no Na^+^ dependence (lanes 7, 8, 11 and 12). Therefore, we conclude that VaFlhA forms the PMF‐driven export gate complex along with the *Salmonella* FlhB, FliO, FliP, FliQ, and FliR at the flagellar base.

### *Vibrio* FlhA fails to exert its export activity in the absence of FliH and FliI

**Figure 3:**
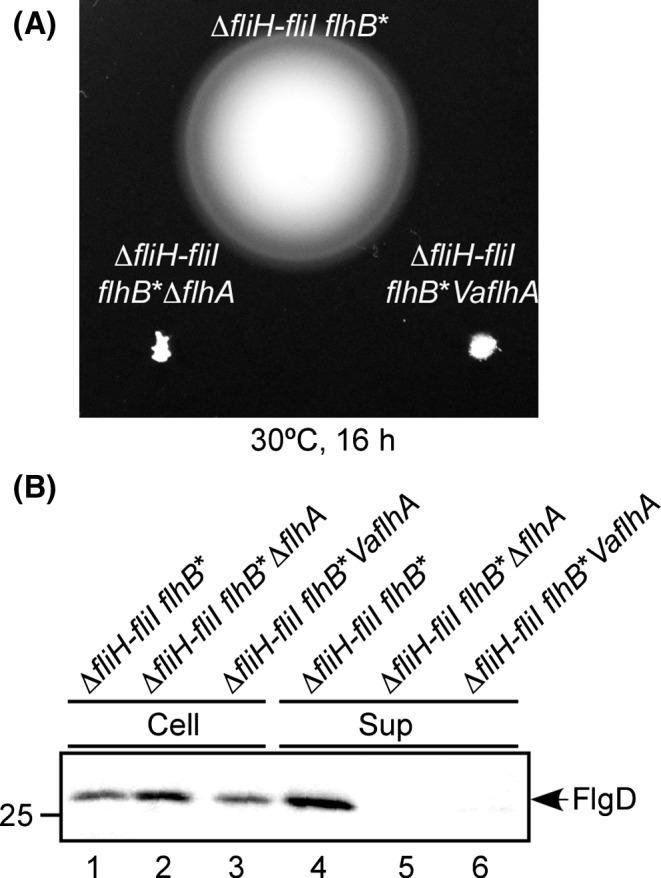
Cross‐complementation assay of *Vibrio* FlhA in the absence of FliH and FliI. (A) Motility of NH004 transformed with pNY101 (∆fliH‐fliI flhB* *VaflhA*) in soft agar. MMHI0117 carrying pSU41 (∆fliH‐fliI flhB*) and NH004 harboring pSU41 (∆fliH‐fliI flhB* ∆*flhA*) were used as the positive and negative controls. Plates were incubated at 30°C for 16 h. (B) Immunoblotting, using polyclonal anti‐FlgD antibody, of whole‐cell proteins (Cell) and culture supernatant fractions (Sup) prepared from the above strains.

Most of mutations at conserved charged residues within FlhA_TM_ were tolerated in the presence of FliH and FliI, but resulted in loss‐of‐function in their absence (Hara et al. 2011, 2012), suggesting that FliH and FliI are critical for the robustness of FlhA against genetic perturbations. To test whether VaFlhA is still functional in the absence of FliH and FliI, we used a Δ*fliH‐fliI flhB(P28T)* bypass mutant whose second‐site P28T mutation in FlhB considerably increases the probability of flagellar protein export in the absence of FliH and FliI (Minamino and Namba 2008). VaFlhA did not restore motility of the *Salmonella* Δ*fliH‐fliI flhB*(P28T) Δ*flhA* mutant (Fig. 3A). In agreement with this, immunoblotting with anti‐FlgD antibody revealed that the Δ*fliH‐fliI flhB*(P28T) *VaflhA* cells did not secrete FlgD into the culture supernatant (Fig. 3B, lane 6). These results indicate that VaFlhA cannot work in this bypass mutant background.

We next investigated the effect of the *flhB*(P28T) mutation by itself on the function of VaFlhA (Fig. S4). VaFlhA fully restored motility of a *Salmonella flhB*(P28T) Δ*flhA* mutant (Fig. S4A). Consistently, FlgD was detected in the culture supernatant of the *flhB(P28T) VaflhA* strain (Fig. S4B, lane 6). These results suggest that the presence of FliH and FliI allows VaFlhA to exert its export function considerably even in the presence of the FlhB(P28T) bypass mutation.

### Isolation of pseudorevertants from the Δ*fliH‐fliI flhB(P28T) VaflhA* strain

**Figure 4:**
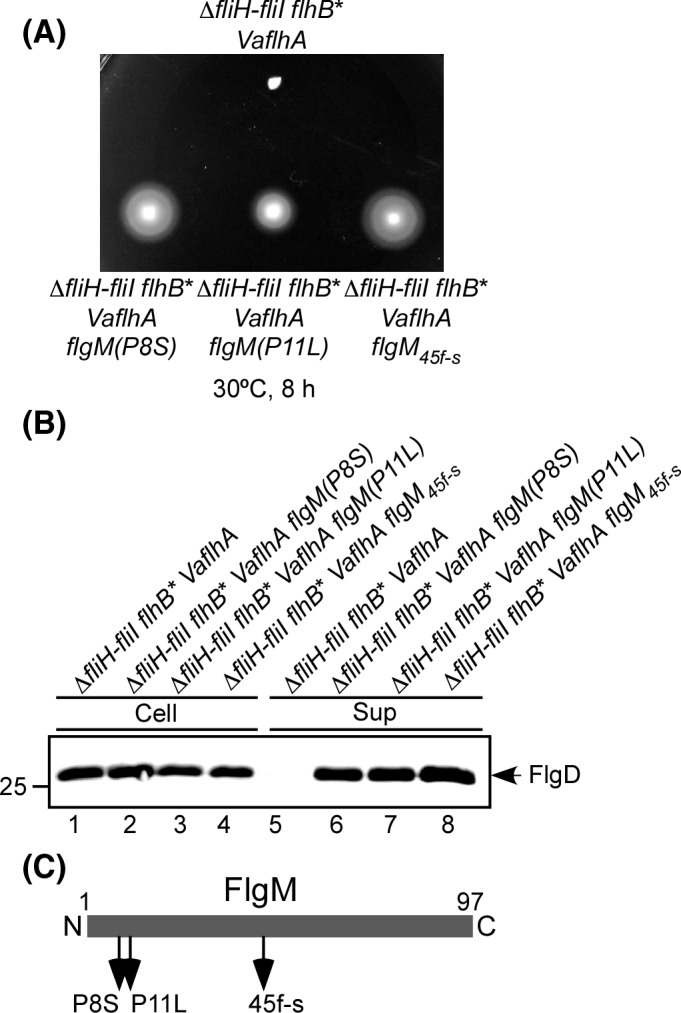
Isolation of pseudorevertants from the **∆** *fliH‐fliI flhB(P28T) VaflhA* strain. (A) Motility of NH004 transformed with pNY101 (∆fliHI flhB* *VaflhA*) and its pseudorevertants, MMA2002 (∆fliH‐fliI flhB* *VaflhA flgM(P8S)*), MMA2003 (∆fliH‐fliI flhB* *VaflhA flgM(P11L)*), and MMA2004 (∆fliH‐fliI flhB* *VaflhA flgM*_*45f‐s*_) at 30°C for 8 h. (B) Immunoblotting, using polyclonal anti‐FlgD antibody, of whole‐cell proteins (Cell) and culture supernatant fractions (Sup) prepared from the above strains. (C) Location of extragenic *flgM* suppressor mutations. FlgM consists of 97 amino acid resides. The sites of suppressor mutations in FlgM are shown by arrows. Missence mutations are indicated as P8S and P11L. A frameshift mutation is indicated by f‐s. The N and C termini of FlgM are labeled as 1 and 97, respectively.

To understand why VaFlhA requires the support of FliH and FliI for its PMF‐driven export activity in *Salmonella*, pseudorevertants were isolated from the Δ*fliH‐fliI flhB*(P28T) *VaflhA* strain by streaking an overnight culture out on soft agar plates, incubating them at 30°C for 2 days and looking for motility halos emerging from the streak. In total, seven motile colonies were purified from such halos. The motility of these pseudorevertants was considerably better than that of the parent strain (Fig. 4A). In agreement with this, FlgD was detected in the culture supernatants of these pseudorevertants (Fig. 4B). DNA sequencing revealed that all suppressor mutations are located in the *flgM* gene (Fig. 4C). They can be divided into two categories. The first category consists of missense mutations: P8S and P11L (isolated four times). The other category is a frameshift at codon 45 (isolated twice), resulting in truncation of the C‐terminal region of FlgM. FlgM acts as an anti‐sigma factor of the flagellar regulon (Gillen and Hughes 1991; Ohnishi et al. 1992). C‐terminal truncations of FlgM cause a loss of its anti‐sigma factor activity (Iyoda and Kutsukake 1995) and hence result in a two‐ to threefold increase in the number of the flagella per cell (Kutsukake and Iino 1994). Because it has been shown that deletions of the cytoplasmic FliH‐FliI‐FliJ ATPase complex can be bypassed by a significant increment in flagellar gene expression levels (Erhardt et al. 2014), we suggest that these *flgM* suppressor mutations considerably increased the cytoplasmic levels of both export substrates and cytoplasmic export proteins, allowing the Δ*fliH‐fliI flhB*(P28T) *VaflhA* strain to export flagellar proteins to produce flagella to considerable degree.

### VaFlhA_L‐C_ requires FliH and FliI for efficient interactions with FliJ and the chaperone–substrate complexes

**Figure 5:**
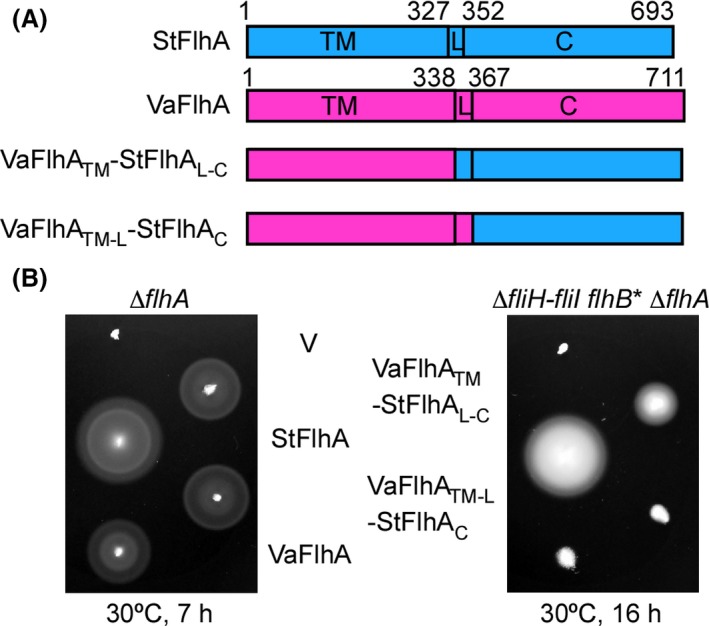
Complementation of FlhA chimera proteins. (A) Representation of the VaFlhA_TM_‐StFlhA_L__‐C_ and VaFlhA_TM__‐L_‐StFlhA_C_ chimera proteins comprising the N‐terminal region of VaFlhA_TM_ (residues 1– 338 of *Vibrio* FlhA) fused to the C‐terminal cytoplasmic region of StFlhA_L__‐C_ (residues 328–693 of *Salmonella* FlhA) and the N‐terminal region of VaFlhA_TM__‐L_ (residues 1–366 of *Vibrio* FlhA) fused to the C‐ cytoplasmic domain of StFlhA_C_ (residues 352–693 of *Salmonella* FlhA), respectively. (B) Motility of NH001 (∆*flhA*) and NH004 (∆fliH‐fliI flhB* ∆*flhA*) transformed with pSU41 (V), pMM130 (StFlhA), pNY101 (VaFlhA), pMKMHA003 (VaFlhA_TM_‐StFlhA_L__‐C_), or pMKMHA004 (VaFlhA_TM__‐L_‐StFlhA_C_) in soft agar at 30°C.

FlhA consists of three regions: FlhA_TM_, FlhA_L_, and FlhA_C_ (Fig. 5A and Fig. S1). Cooperative interactions of FlhA_TM_ with FlhB, FliH, FliI, and FliR are required for the translocation of export substrates in a PMF‐dependent manner. FlhA_L_ connecting FlhA_C_ to FlhA_TM_ is responsible for the interaction with FliJ (Bange et al. 2010; Minamino et al. 2011), and the D1 and D2 domains of FlhA_C_ are directly involved in interactions with the flagellar chaperone–substrate complexes (Bange et al. 2010; Minamino et al. 2012a; Kinoshita et al. 2013). To identify which regions of VaFlhA require support of FliH and FliI to interact with its binding partners, we replaced VaFlhA_L_ and VaFlhA_C_ by StFlhA_L_ and StFlhA_C_, respectively, to create two FlhA chimeras, VaFlhA_TM‐L_‐StFlhA_C_ and VaFlhA_TM_‐StFlhA_L‐C_ (Fig. 5A). These two chimeric proteins conferred motility of the *Salmonella* Δ*flhA* mutant in a way similar to VaFlhA (Fig. 5B, left panel), indicating their capability of forming the export gate complex along with other *Salmonella* export gate proteins. The VaFlhA_TM_‐StFlhA_L‐C_ chimeric protein restored motility of the Δ*fliH‐fliI flhB*(P28T) Δ*flhA* mutant to some degree, whereas the VaFlhA_TM‐L_‐StFlhA_C_ did not at all (Fig. 5B, right panel). These results indicate that VaFlhA_L‐C_ requires FliH and FliI to efficiently and properly interact with its binding partners.

**Figure 6:**
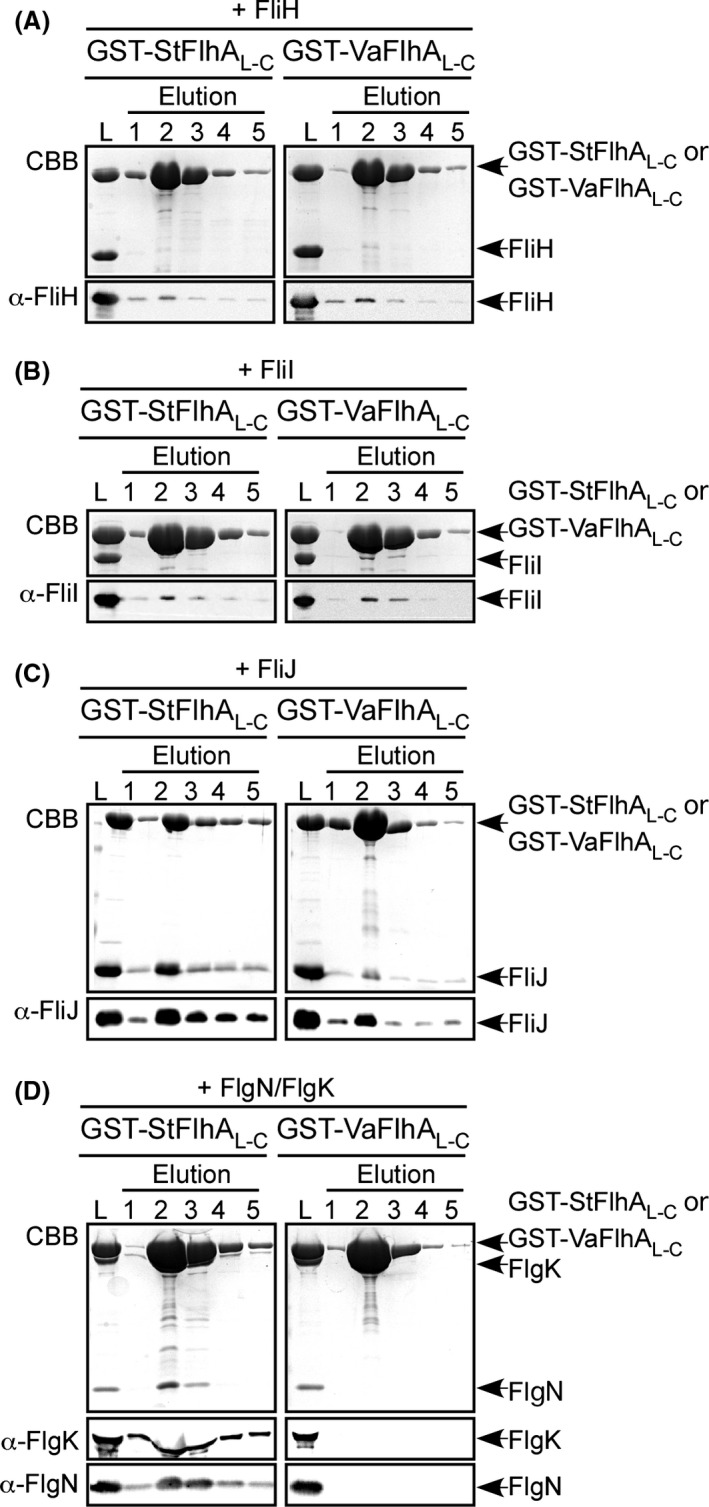
Interaction of *Vibrio* FlhA with FliH, FliI, FliJ, and the flagellar chaperone–substrate complex. Purified (A) FliH, (B) FliI, (C) FliJ, or (D) FlgN/FlgK complex was mixed with purified GST‐StFlhA_L__‐C_ (left panel) or GST‐VaFlhA_L__‐C_ (right panel), and dialyzed overnight against PBS. These mixtures (L) were loaded onto a GST column. After washing with 10 mL PBS, proteins were eluted with 10 mmol/L reduced glutathione. Elution fractions were analyzed by Coomassie Brilliant blue (CBB) staining (first rows) and immunoblotting by polyclonal anti‐FliH (A, second rows), anti‐FliI (B, second rows), anti‐FliJ (C, second rows), anti‐FlgK (D, second rows) or anti‐FlgN antibody (D, third rows).

To analyze the binding affinities of VaFlhA_L‐C_ for FliH, FliI, FliJ, and the FlgN–FlgK chaperone–substrate complexes, we carried out pull‐down assays by GST affinity chromatography (Fig. 6). GST‐VaFlhA_L‐C_ bound to FliH and FliI at levels similar to GST‐StFlhA_L‐C_ (Fig. 6A and B), indicating that the binding affinities of VaFlhA_L‐C_ for FliH and FliI were essentially the same as those of StFlhA_L‐C_. The amount of FliJ co‐purified with GST‐VaFlhA_L‐C_ was about 10‐fold lower than those co‐purified with GST‐StFlhA_L‐C_ (Fig. 6C). The FlgN–FlgK complex co‐purified with GST‐StFlhA_L‐C_ but not with GST‐VaFlhA_L‐C_ (Fig. 6D). These observations indicate that the binding affinities of VaFlhA_L‐C_ are weaker for FliJ and markedly weaker for the FlgN/FlgK complex than those of StFlhA_L‐C_. Neither FliH, FliI, FliJ nor the FlgN–FlgK complex co‐purified with GST (data not shown), in agreement with previous reports (Minamino et al. 2010, 2012a,b). These results confirmed that the low affinity of VaFlhA_L‐C_ for FliJ and the FlgN/FlgK complex are somehow compensated by FliH and FliI.

## Discussion

The flagellar type III export apparatus transports 14 flagellar proteins with their copy numbers ranging from a few to a few tens of thousands during flagellar assembly. A remarkable feature of type III protein export is that the export apparatus can coordinate protein export with assembly. Therefore, flagellar type III protein export is a complex process involving a substantial number of checkpoints to ensure the correct order of export (Chevance and Hughes 2008; Minamino 2014). A plausible export mechanism has been proposed. The cytoplasmic ATPase complexes consisting of FliH, FliI, and FliJ bind to export substrates and chaperone–substrate complexes in the cytoplasm and deliver the substrates and chaperone–substrate complexes to the base of the growing flagellar structure through interactions of FliH with FlhA and FliN (Bai et al. 2014). Once ATP hydrolysis by FliI ATPase activates a PMF‐driven TM export gate complex through an interaction between FliJ and FlhA, the export gate processively transports the substrates in a PMF‐dependent manner (Minamino et al. 2014).

**Figure 7:**
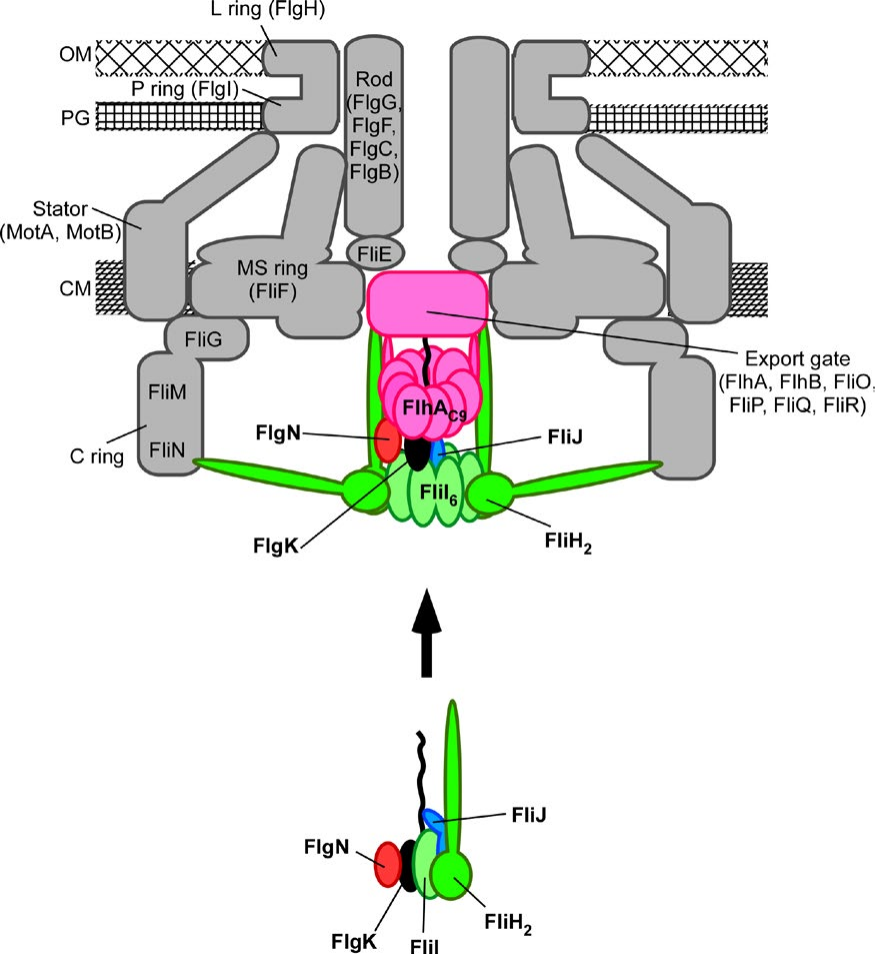
Schematic diagram of the flagellar type III protein export apparatus. The export gate is composed of six transmembrane proteins, FlhA, FlhB, FliO, FliP, FliQ, and FliR and is located within the MS ring. The C‐terminal cytoplasmic domain of FlhA (FlhA_C_) forms part of the docking platform for FliH, FliI ATPase, FliJ, flagellar type III secretion chaperones, and export substrates. FliI forms a hetero‐trimer with the FliH dimer in the cytoplasm. The FliH_2_–FliI complex acts as a dynamic carrier to deliver FliJ and the FlgN–FlgK chaperone‐export substrate complex to the FlhA_C_ ring complex (FlhA_C__9_). Upon formation of the FliI_6_FliJ ring complex on the FlhA_C__9_ ring, the FliI_6_FliJ ring complex hydrolyses ATP and activates the export gate through an interaction between FliJ and FlhA, allowing the gate to translocate FlgK into the central channel of the growing flagellar structure. OM, outer membrane; PG, peptidoglycan layer; CM, cytoplasmic membrane.

The cytoplasmic ATPase complex shares an evolutionary relationship with F‐ and V‐type rotary ATPases (Pallen et al. 2006; Imada et al. 2007; Ibuki et al. 2011; Kishikawa et al. 2013). An increase in the cytoplasmic levels of export substrates and an increment in PMF are capable of bypassing the absence of the cytoplasmic FliH–FliI–FliJ ATPase complex (Erhardt et al. 2014). Biological systems can generally maintain their functional activities against internal and external perturbations. Since such robustness is a fundamental property that biological systems have evolved to gain by natural selection (Kitano 2004), it has been proposed that environmental pressures have driven the flagellar type III export apparatus to evolve via distinct evolutionary steps to develop robustness and efficiency by late addition of the cytoplasmic ATPase complex (Erhardt et al. 2014). However, it remained unknown how the ATPase complex ensures efficiency and robustness of the flagellar export system against various perturbations. In this study, we carried out a cross‐complementation assay to examine mutational robustness of an energy transduction system of the flagellar type III export apparatus. We found that VaFlhA is functional in *Salmonella* in the presence of FliH and FliI but not in their absence (Figs. 1, 3). We also showed that *flgM* mutations allow VaFlhA to function in the *Salmonella* export system even in the absence of FliH and FliI (Fig. 4). Because a loss of FlgM results in a considerable increment in the expression levels of all flagellar genes (Kutsukake and Iino 1994), we suggest that an increase in the cytoplasmic levels of FliJ, flagellar chaperones, and export substrates allows the export gate complex containing VaFlhA to transport export substrates into the distal end of the growing structure in the absence of FliH and FliI. The binding affinities of VaFlhA_L‐C_ for FliJ and FlgN–FlgK chaperone substrate complex are much lower than those of StFlhA_L‐C_ (Fig. 6). Therefore, we suggest that FliH and FliI ensure efficient recruitment of FliJ, flagellar chaperones, and export substrates to their binding sites of FlhA_L‐C_ (Fig. 7).

FliI ATPase exists not only as the FliI_6_ ring stably bound within the C ring to fully exert its ATPase activity but also as the FliH_2_FliI complex in the cytoplasm (Fig. 7) (Minamino and Macnab 2000b; Chen et al. 2011; Kawamoto et al. 2013; Bai et al. 2014). The chaperone–substrate complexes bind to the FliH_2_FliI complex through cooperative interactions between FliI and chaperone (Thomas et al. 2004; Imada et al. 2010; Minamino et al. 2012b). FliJ interacts not only with the central cavity of the FliI_6_ ring (Ibuki et al. 2011) but also with the FliH_2_FliI complex through cooperative interactions among FliH, FliI, and FliJ (González‐Pedrajo et al. 2002). Since FliI‐YFP shows dynamic turnovers between the basal body and the cytoplasmic pool as observed by fluorescence recovery after photobleaching of FliI‐YFP spots, the FliH_2_FliI complex is thought to act as a dynamic carrier to deliver FliJ and the chaperone–export substrate complexes to the docking platform formed by FlhA_C_ (Bai et al. 2014). FliJ and the chaperone–substrate complexes bind to FlhA_L_ and a well‐conserved hydrophobic dimple located at the interface between domains D1 and D2 of FlhA_C_, respectively (Bange et al. 2010; Minamino et al. 2011, 2012a; Kinoshita et al. 2013). Domain D2 of FlhA_C_ is directly involved in the translocation of the export substrate (Minamino et al. 2010; Shimada et al. 2012). Because an interaction between FliJ and FlhA brought about by FliH and FliI allows the export gate to efficiently utilize PMF to facilitate flagellar protein export (Minamino et al. 2011; Ibuki et al. 2013), we propose that FliH and FliI contribute to an efficient and robust energy coupling mechanism of flagellar type III protein export during flagellar assembly by promoting and enforcing the interactions between FlhA and its partner proteins (Fig. 7).

## Conflict of interest

None declared.

## Supporting information


**Figure S1**. Sequence alignment of FlhA proteins of *Salmonella enterica* and *Vibrio alginolyticus*.**Figure S2.** Characterization of a *Salmonella VaflhA* strain.**Figure S3.** Effects of (A) carbonyl cyanide *m*‐chlorophenylhydrazone (CCCP) and (B) 100 mmol/L NaCl on the level of FlgD secreted by the *VaflhA* cells.**Figure S4.** Effect of the FlhB(P28T) mutation on motility of the *VaflhA* cells.
