# Supplementary material for: FliH and FliI ensure efficient energy coupling of flagellar type III protein export in Salmonella
Source: Microbiologyopen. 2016 Feb 25;5(3):424–35. doi: 10.1002/mbo3.340 (PMC4905995; doi:10.1002/mbo3.340)
Supplement: Supplementary file 1 — Figure S1. Sequence alignment of FlhA proteins of Salmonella enterica and Vibrio alginolyticus.Figure S2. Characterization of a Salmonella VaflhA strain.Figure S3. Effects of (A) carbonyl cyanide m‐chlorophenylhydrazone (CCCP) and (B) 100 mmol/L NaCl on the level of FlgD secreted by the VaflhA cells.Figure S4. Effect of the FlhB(P28T) mutation on motility of the VaflhA cells. [file MBO3-5-424-s001.pdf]

Supporting Information

FliH and FliI ensure efficient energy coupling of flagellar type III protein export in *Salmonella*

Tohru Minamino, Miki Kinoshita, Yumi Inoue, Yusuke V. Morimoto, Kunio Ihara, Satomi Koya, Noritaka Hara, Noriko Nishioka, Seiji Kojima, Michio Homma and Keiichi Namba

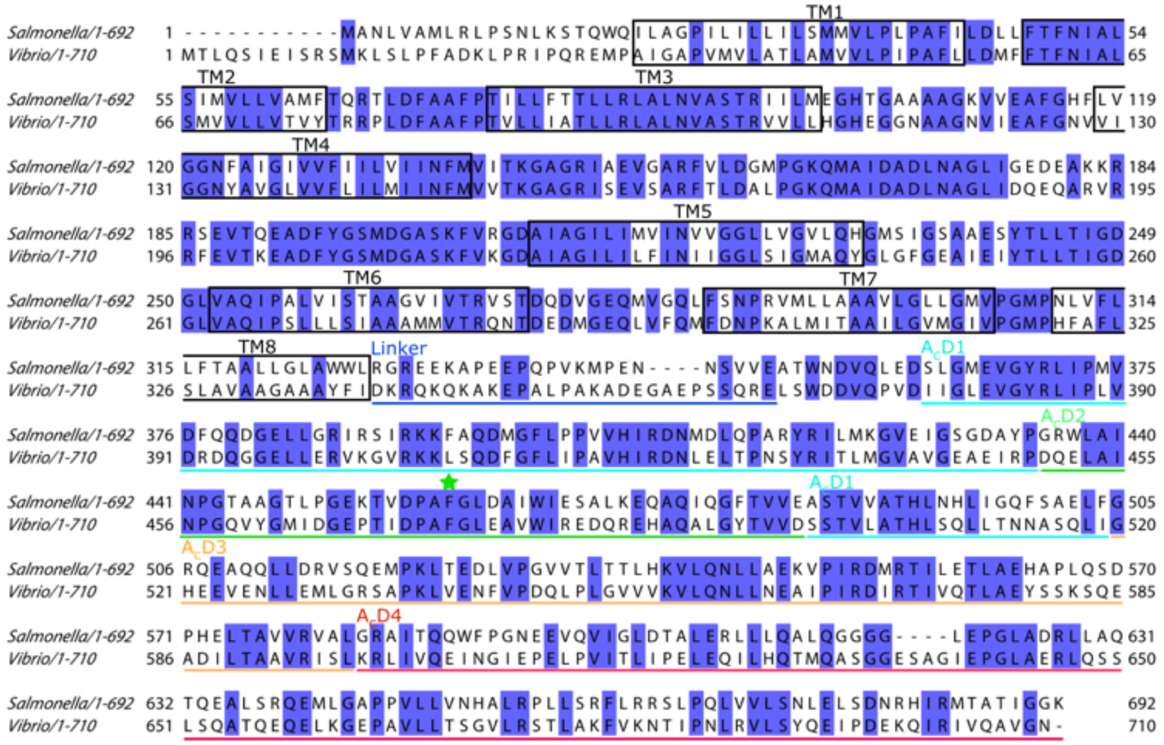

Figure S1. Sequence alignment of FlhA proteins of *Salmonella enterica* and *Vibrio alginolyticus*. Sequence alignment was carried out by Clustal Omega. FlhA consists of three regions: $FlhA_{TM}$ with eight predicted transmembrane helices (TM1 to TM8), a flexible linker and $FlhA_C$ consisting of four domains (D1, D2, D3 and D4). Green star indicates a well-conserved Phe residue responsible for the interaction of FlhA with the flagellar chaperone-substrate complexes. The linker region of FlhA is responsible for the interaction with FliJ.

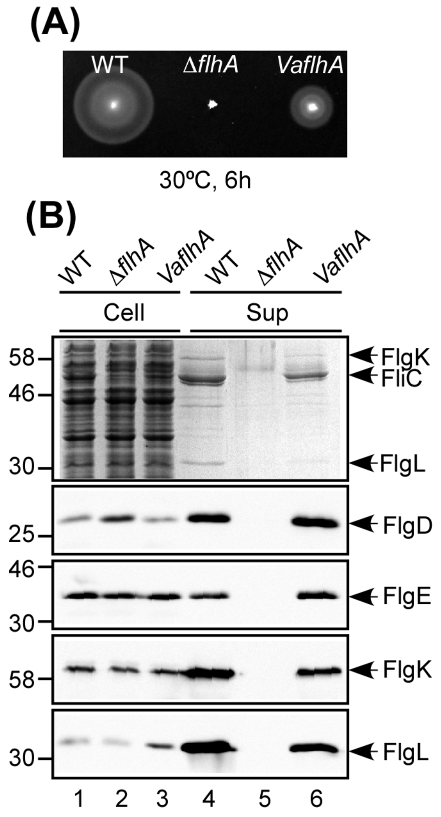

Figure S2. Characterization of a *Salmonella VaflhA* strain. (A) Motility of SJW1103 (WT), NH001 (Δ*flhA*) and MMA2001 (*VaflhA*) in soft agar. The *flhA* gene on the chromosome was replaced by the Vibrio *flhA* gene. Plates were incubated at 30 ºC for 6 hours. (B) Secretion assays. Whole cell proteins (Cell) and culture supernatant fractions (Sup) were prepared from the above strains, and then analyzed by CBB staining (1st row) and immunoblotting, using polyclonal anti-FlgD (2nd row), anti-FlgE (3rd row), anti-FlgK (4th row) or anti-FlgL (5th row) antibody. The positions of molecular mass markers are indicated on the left.

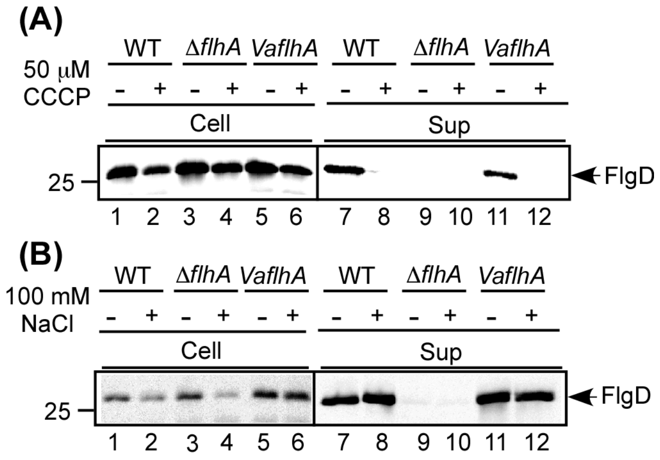

Figure S3. Effects of (A) CCCP and (B) 100 mM NaCl on the level of FlgD secreted by the *VaflhA* cells. (A) Immunoblotting, using polyclonal anti-FlgD antibody, of whole cell proteins (Cell) and culture supernatant fractions (Sup) prepared from SJW1103 carrying pSU41 (WT), NH001 harbouring pSU41 (Δ*flhA*) and NH001 transformed with pNY101 (*VaflhA*) grown in the presence and absence of 50 μM CCCP. (B) Immunoblotting, using polyclonal anti-FlgD antibody, of whole cell proteins (Cell) and culture supernatant fractions (Sup) prepared from SJW1103 carrying pSU41 (WT), NH001 harbouring pSU41 (Δ*flhA*) and NH001 transformed with pNY101 (*VaflhA*) grown in the presence and absence of 100 mM NaCl.

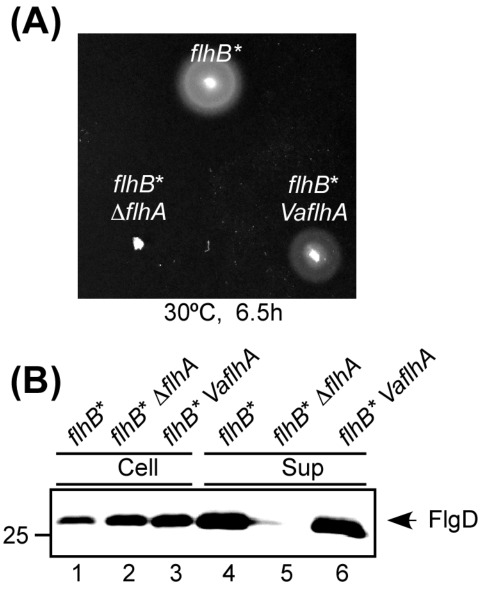

Figure S4. Effect of the FlhB(P28T) mutation on motility of the *VaflhA* cells. (A) Motility of MMB017 carrying pSU41 (*flhB**), NH002 harboring pSU41 (*flhB** Δ*flhA*) and NH002transformed with pNY101 (*flhB** *VaflhA*) in soft agar. Plates were incubated at 30 ºC for 6.5 hours. (B) Secretion assays. Immunoblotting, using polyclonal anti-FlgD antibody, of whole cell proteins (Cell) and culture supernatant fractions (Sup) prepared from the same transformants.
